# Models for gene duplication when dosage balance works as a transition state to subsequent neo-or sub-functionalization

**DOI:** 10.1186/s12862-016-0616-1

**Published:** 2016-02-20

**Authors:** Ashley I. Teufel, Liang Liu, David A. Liberles

**Affiliations:** Department of Biology and Center for Computational Genetics and Genomics, Temple University, Philadelphia, PA 19122 USA; Department of Molecular Biology, University of Wyoming, Laramie, WY 82071 USA; Department of Statistics, University of Georgia, Athens, GA 30602 USA

**Keywords:** Duplicate gene retention, Protein complex, Functional change, Probabilistic model, Birth-death process

## Abstract

**Background:**

Dosage balance has been described as an important process for the retention of duplicate genes after whole genome duplication events. However, dosage balance is only a temporary mechanism for duplicate gene retention, as it ceases to function following the stochastic loss of interacting partners, as dosage balance itself is lost with this event. With the prolonged period of retention, on the other hand, there is the potential for the accumulation of substitutions which upon release from dosage balance constraints, can lead to either subsequent neo-functionalization or sub-functionalization. Mechanistic models developed to date for duplicate gene retention treat these processes independently, but do not describe dosage balance as a transition state to eventual functional change.

**Results:**

Here a model for these processes (dosage plus neofunctionalization and dosage plus subfunctionalization) has been built within an existing framework. Because of the computational complexity of these models, a simpler modeling framework that captures the same information is also proposed. This model is integrated into a phylogenetic birth-death model, expanding the range of available models.

**Conclusions:**

Including further levels of biological reality in methods for gene tree/species tree reconciliation should not only increase the accuracy of estimates of the timing and evolutionary history of genes but can also offer insight into how genes and genomes evolve. These new models add to the tool box for characterizing mechanisms of duplicate gene retention probabilistically.

## Background

The duplication of genes provides a platform for evolution to act upon and is believed to be a major source of structural and functional divergence in genome evolution [1–3]. This is because, according to the classic theory, gene duplication events provide a source of genetic material that is freed from the selective pressures experienced by the unduplicated original copy, allowing for the duplicated genes to freely accumulate changes. Large increases in the number of genes have been coupled to expansions in organismal complexity and diversification in metazoans and angiosperms [4, 5].

While the ultimate fate of duplicated genes from larger-scale duplication events involving multiple interacting partners is often determined by mutations resulting in functional change [6] stoichiometric constraints are expected to influence the length of evolutionary time that networks of interacting genes are preserved [7, 8]. This is because misbalance of the concentration of interacting partners can lead to large concentrations of exposed hydrophobic patches, with the ability to potentially negatively influence fitness through improper protein complex assembly, spurious interactions, or deleterious downstream effects on pathways [9, 10]. One would expect that duplicates maintained under stoichiometric constraint would be preserved for longer evolutionary timescales due to selection against the loss of interacting partners, though once the network has been perturbed, the remaining members of the network would be quickly lost. This prolonged initial retention due to dosage constraints increases the length of time the mutational process has to act on duplicated genes and is thought to be an intermediate step to retention by neo- or subfunctionalization [11–15]. Selection against duplicates due to gene expression costs has not been considered in these models [16].

The dosage balance mechanism is most relevant to whole genome duplication events, where every gene in the genome is duplicated. It may also be applicable to tandem duplication events in genes in operons, where linked genes function together. Genes originating in a smaller scale duplication event that do not have interacting partners (for example enzymes that function as monomers) would not be subjected to this model whereas for those with interacting partners, a positive selective pressure for loss might occur [13, 15].

Models for duplicate gene retention can give insight into how genes and genomes functionally diverge along lineages of a species tree. These models can be applied in pairwise analysis of recent duplicates in a genome to characterize the average properties of synonymous substitution rate (dS)-dependent duplicate gene retention [17–19] and can also be incorporated into phylogenetic contexts [20]. From datasets such as these, information about the instantaneous rate of loss of duplicated genes over evolutionary time can be used to make inference about the mechanism of duplicate gene retention, as each mechanism of retention has a distinct time-dependent loss rate (hazard).

Konrad et al. [13] characterizes these loss rates for neofunctionalization and subfunctionalization when considered independently of the influence of stoichiometric constraints. In the case of neofunctionalization, where a collection of duplicate genes gains a novel function while their paralogs maintain the ancestral function, the rate of loss is initially high and then decreases as adaptive substitutions are introduced. Averaging over the distribution of waiting time for these adaptive substitutions gives a hazard function which decays convexly to a lower asymptotic rate. Subfunctionalization is a process by which multiple members of a duplicated genes acquire complementary partial loss of function mutations, such that they must be retained together to perform the ancestral function. This is characterized by an instantaneous rate of loss similar to that of neofunctionalization, although it decays concavely due to the extending waiting time for multiple changes. The instantaneous loss rate of duplicates which have lost functionality (nonfunctionalized) remains constant over evolutionary time. Duplicate genes under stoichiometric constraints are expected to have a low instantaneous rate of loss. As members of the complex are lost, the hazard rate increases. This reflects an averaging over the distribution of waiting times for loss events and leads to a hazard function which increases exponentially. It is unclear if there is ever signal for strong positive (cooperative) selective pressure for loss following loss of interacting partners, or if the hazard correspondingly becomes larger than the nonfunctionalization hazard rate [13]. Perhaps the baseline rate accounts for the probability of small scale duplicates that are introduced without interacting partners.

With sets of models for duplicate gene retention following gene duplication events, phylogenetic birth-death models can be developed [15, 21] These models are useful for the process of gene tree-species tree reconciliation [22] that enable mapping of gene tree lineages to the species tree lineages in which they evolved and simultaneous probabilistic inference of the accompanying model that best explained the patterns of retention. It is with a full set of models integrated into such a framework that biological comparative genomic data can be evaluated.

Here, we introduce a new model which incorporates the dynamics of genes that initially experience retention due to dosage constraints, but which are ultimately retained via the processes of neo- or sub-functionalization. This model explicitly assumes that dosage balance ends before the process leading to retention due to neo- or sub-functionalization begins and this assumption is particularly reasonable when dosage constraints prevent the accumulation of substitutions that would lead to functional shifts. Following the development of the new model, its incorporation into a phylogenetic birth-death model is presented.

## Methods

### Model fitting

At various points in this manuscript, data produced from one distribution was fit by another distribution. Expectations of the survival function were generated (at intervals of 0.01 with 30 total data points). Fit of a different model was optimized by minimizing the sum of squares of the values generated from each distribution using differential evolution. This was accomplished with the use of the DEoptim library in R [23].

## Results and discussion

### Model

Previously, a model to account for the mechanistic properties of duplicate gene retention has been described in terms of a hazard function [13].

1 $$ \lambda (t)=f{e}^{-b{t}^c}+d $$

Here λ(t) is the hazard function describing the instantaneous rate of loss, b is a scaling parameter and the f and d parameters allow for loss from d + f to the asymptote d. A hazard rate corresponding to the dynamics of neofunctionalization is described when 0 < c < 1, and subfunctionalization when c > 1. Nonfunctionalization is defined using just d as a constant rate. Dosage balance can be defined when b < 0 and d = −f. The characteristic exponential curve which describes the process of dosage balance is representative of averaged effects of the times in which duplicated genes began to experience an increased hazard rate.

When dosage balance is acting as a mechanism in tandem with neo- or subfunctionalization, the dynamics experienced by duplicated genes once they are no longer being maintained under dosage balance and begin to experience an increased level of hazard that can be described by either the sub- or neofunctionalization models. In this case, the hazard rate for individual duplicate pairs in stoichiometric balance is treated as *π*, which corresponds to the averaged rate y-intercept of *π*=0 in the model from [13]. For simplicity, genes out of stoichiometric balance are not assumed to have any additional hazard beyond that of mutation-driven non-functionalization.

The fraction of genes experiencing retention due to dosage balance immediately after duplication which will eventually experience retention dynamics characteristic of neo- or subfunctionalization can be expressed as

2 $$ \omega (t)=1-\frac{ \min\ \left(\lambda {(t)}_{dos},\kern0.75em {d}^{\hbox{'}}+{f}^{\hbox{'}}\right)}{d^{\hbox{'}}+{f}^{\hbox{'}}} $$

Here, *λ*(*t*)_*dos*_ represents the hazard function parameterized for dosage balance dynamics where b < 0 and d = −f gives a low rate of gene loss due to stoichiometric constraints immediately experienced after duplication. d’ and f’ correspond to the d and f parameters in *λ*(*t*) when the model is parameterized for either neo- or sub-functionalization retention mechanisms denoted as *λ*(*t*)_*neo*/*sub*_. These give a high loss rate immediately experienced after duplication when genes are not being preserved due to selection (mutation-driven non-functionalization). The prime notation is introduced to distinguish between parameters which correspond to *λ*(*t*)_*dos*_ and parameters which correspond to *λ*(*t*)_*neo*/*sub*_ when two parameterizations of *λ*(*t*) are necessary to describe a composite of loss dynamics. As indicated, this formulation captures the fraction of genes that are initially retained due to dosage balance after duplication. When interacting partners are lost, the effectiveness of the dosage constraints to maintain genes is lost. For an individual gene, when the dosage mechanism stops acting, the loss rate of the gene escapes protection due to dosage balance and experiences loss characteristic of the initial dynamics of neo- or sub-functionalization (d’ + f’) starting from a neutral rate. This is a feature of the model as it is described mathematically, but may or may not be true biologically. Alternative assumptions of the biology of the hybrid process can also be described mathematically and the work here presents an initial description.

The composite hazard function for the mixture of dosage balance and other retention mechanisms is expressed as

3 $$ \delta (t)=\frac{\omega (t)}{\rho (t)}\pi +{\displaystyle {\int}_0^t\frac{1-\omega (y)}{\rho (t)}\lambda {(y)}_{neo/sub} dy} $$

**Fig. 1:**
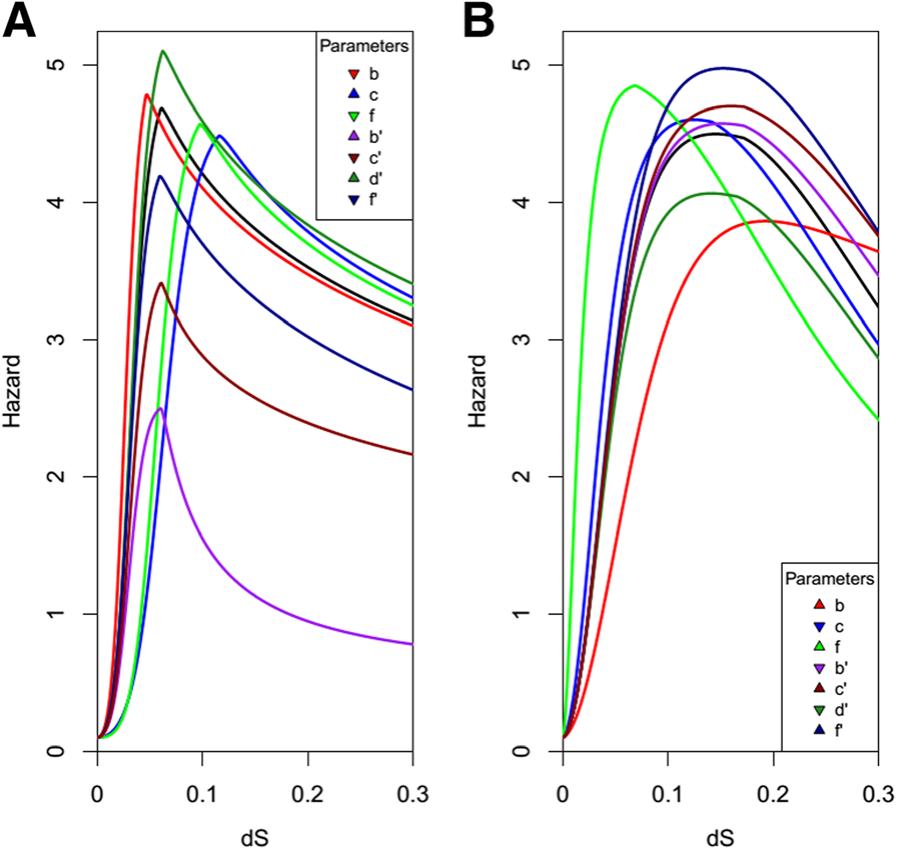
Examples of composite hazard shapes. **a** Composite hazard created by mixing dosage balance and neofunctionalization dynamics. The black is determined by the parameter values b = −35, c = .5, f = 0.001, b’ = 2, c’ = 0.5, d’ = 5, f’ = 0.5, each colored line shows a deviation in a single parameter value. b = −40 (*red*), c = 0.65 (*blue*), f = 0.0001 (*green*), b’ = 12 (*purple*), c’ = 0.25 (*maroon*), d’ = 5.5 (*forest green*), f’ = 0 (*navy blue*). Arrows in the legend indicate if the change in parameter value is an increase or decrease compared to the initial values represented by the black line. **b** Composite hazard created by mixing dosage balance and subfunctionalization dynamics. The black is determined by the parameter values b = −15, c = 0.5, f = 0.01, b’ = 50, c’ = 2, d’ = 5, f’ = 0, each colored line shows a deviation in a single parameter value. b = −10 (*red*), c = 0.45 (*blue*), f = 0.1 (*green*), b’ = 40 (*purple*), c’ = 2.25 (*maroon*), d’ = 4.5 (*forest green*), f’ = 0.5 (*navy blue*). Arrows in the legend indicate if the change in parameter value is an increase or decrease compared to the initial values represented by the black line. The illustrative deviations in parameter values were chosen to be consistent relative to the initial values to visually demonstrate the scale of influence that each parameter has on the curve shape. In summary, b, c, and f are the parameters of the dosage Weibull distribution, where b is the scale parameter, c is the shape parameter, and f is the overall scalar of the transition. For the neofunctionalization and subfunctionalization components, b’ is the scale parameter, c’ is the shape parameter, d’ + f’ determine the initial hazard when dosage transitions to decay, and d’ reflects the hazard rate for non-redundant genes as an asymptote. The dosage parameters (b, c, and f) characterize the initial increase in the hazard whereas the prime parameters (b’, c’, d’, and f’) reflect the decay process as genes are either lost or differentially functionalized

Here *ρ*(*t*) = *ω*(*t*) + ∫_0_^*t*^1 − *ω*(*x*)*dx* serves as a normalization factor for the fraction of the genes experiencing retention due to dosage balance (*ω*(*t*)) and *π* is the constant low hazard rate experience by these genes. The normalization factor (*ρ*(*t*)) is introduced so that the total fraction of genes experiencing retention due to dosage balance plus the fraction of genes experiencing retention due to the neo- or sub-functionalization retention mechanisms sums to 1. x and y are integration indices that track time to enable integration over neo- or sub-functionalization processes that start before current time t. This integration is necessary because the neofunctionalization and subfunctionalization processes are time-dependent functions that are not starting at global t = 0. This composite hazard function can be interpreted as the sum effect of the fraction of genes experiencing a low (zero in this instance) rate of loss (*π*) due to dosage balance and the fraction of genes experiencing loss as function of the length of time (y) neo- or sub-functionalization dynamics were experienced. The exact mathematical formulation is consistent with an initial loss rate of zero, but can be scaled to incorporate nonzero values in the scaling of the fraction under neo- or sub-functionalization constraints. Figure 1 demonstrates various hazard shapes generated by this mixture process of dosage balance and neofunctionalization (Fig. 1a) as well as subfunctionalization (Fig. 1b). These examples demonstrate the influence of consistent changes in parameter values on changes in hazard shape. Each of the colored lines represents variations in a single parameter from a model shown in black. The scaling parameter b (red) in the dosage balance portion of the model (*λ*(*t*)_*dos*_) determines the constant rate of movement from duplicates maintained under dosage constraints to retention due to sub- or neo-functionalization. The exponential term c (blue) determines the relative rate of change in movement of duplicates between experiencing a constant low loss rate to a rate that is dependent on a functional mechanism of retention. This transition process is scaled by f (green), which serves to extend or shorten the cumulative transition rate determined by the combination of the constant rate (b) and the relative rate (c). These together reflect the time-dependent rate at which duplicates involved in protein complexes or sets of interactions, lose the duplicate copies of those interacting partners, such that they are no longer retained at the dosage rate. Mechanistic interpretations of the parameters associated *λ*(*t*)_*neo*/*sub*_ are the same as outlined in [13]. Denoting these parameters with primes, b’ (purple) represents the constant rate of decay. The c’ (forest green) parameter specifies the relative difference in curve shape from the neutral expectation of nonfunctionalization and acts to determine the convexity or concavity indicative of specific retention mechanisms. The terms d’ + f’ (navy blue and maroon) give the initial high hazard rate experienced due to a lack of selective pressure for maintenance. As substitutions occur, the average hazard function decays to a lower level f’ indicative of preservation due to a functional mechanism. Duplicates that lose preservation under the dosage model are instantaneously subjected to the d’ + f’ hazard rate and subsequent hazard rate decay according to the appropriate retention model.

From examining the curve shapes in Fig. 1, it appears that the mixture of the two processes initially produces dynamics similar to the exponential model. However, as the functional mechanism of retention-dependent dynamics becomes a larger fraction of the total dynamics, the exponential-like increase slows and approaches an upper asymptote. Decay from this asymptote then appears to proceed in a manner characteristic of neo- or sub-functionalization dynamics. Considering the computational complexity associated with the mixture hazard outlined above because of the need to evaluate multiple integrals to solve for the expected value at every specific value of time, it is preferable to introduce a simpler model. Using a simpler piecewise function, the dynamics described by the more complex model can be recapitulated in a framework which encapsulates a single instance of a modified version of *λ*(*t*) that has the ability to offer mechanistic insight, and is computationally tractable to allow for efficient evaluation of the survival function.

4 $$ \varphi (t)=\left\{\begin{array}{c}\hfill \frac{d+f}{{\left(1+h{e}^{-jt}\right)}^k},\kern0.5em t<g\hfill \\ {}\hfill f{e}^{-b{\left(t-g\right)}^c}+d,\kern0.5em t\ge g\hfill \end{array}\right. $$

In this model the initial dynamics due to the influence of dosage balance are described by a generalized logistic function. This function increases from h reflective of the lower hazard rate experienced by genes maintained due to dosage constraints, to the upper asymptote d + f indicative of the hazard rate experience by duplicated genes without selective pressures for their retention, at interacting partner loss rate j (reflecting the curve growth rate). The parameter k affects the shape of the transition between the lower dosage-balanced and upper non-functionalization asymptotes, and the parameter h describes the initial hazard rate experienced at *φ*(0). After a point in time g reflecting the transition as a single point, dynamics associated with the functionally dependent mechanisms of retention such as subfunctionalization or neofunctionalization as described in [13] are employed to describe the loss process.

### Characterization of the hybrid process model

In order to test the ability of the piecewise model to recover functional dynamics, hazard data was generated using a discretized version of the weighted mixing of processes with parameter values from Table 1. This was done by expressing the weighting term as a normalized vector

**Table 1:** Values used in the weighted mixture model to simulate survival data and the values recovered by fitting the piece wise model to this data are given

Simulated	b	c	f	π	b’	c’	d’	f’
A. red	−35	0.5	0.001	0.1	2	0.5	5	0
A. blue	−15	0.5	0.01	0.1	2	0.25	5	0
A. green	−40	0.65	0.001	0.001	10	0.75	3	2
B. red	−55	0.75	0.01	0.01	50	2	5	0
B. blue	−25	0.95	0.1	0.001	175	2	5	0
B. green	−55	0.75	0.001	0.1	100	3	4	1
Recovered	j	k	h	g	b	c	d	f
A. red	104.559	0.864	334.138	0.054	1.969	0.573	3.964	1.056
A. blue	32.739	1.250	17.319	0.168	3.206	0.707	1.717	1.165
A. green	70.915	1.494	181.852	0.087	5.999	0.823	2.393	2.311
B. red	172.00	0.771	4490.00	0.053	13.00	1.740	3.660	1.360
B. blue	35.584	2.304	8.846	0.136	14.554	1.354	3.458	0.901
B. green	114.00	0.515	23400.0	0.077	32.10	2.830	2.220	2.770

5 $$ \overrightarrow{\omega_t}=<\omega \left({t}_i\right),\ 1-\omega \left({t}_i\right),\ 1-\omega \left({t}_{i-1}\right),\ 1 - \omega \left({t}_{i-2}\right), \dots, 1-\omega \left({t}_0\right)> $$

Here *i* represents indices of discretized measures of time. *δ*(*t*) can then be expressed as a sum of the fraction of the dynamics experiencing retention due to dosage balance and the fraction of genes experiencing retention due to sub or neofunctionalization dynamics as

6 $$ \delta (t)=\overrightarrow{\omega_t}\left[0\right]\ \pi +{\displaystyle {\sum}_{j=0}^t\overrightarrow{\omega_t}\left[j+1\right]\lambda {\left({t}_j\right)}_{neo/sub}} $$

**Fig. 2:**
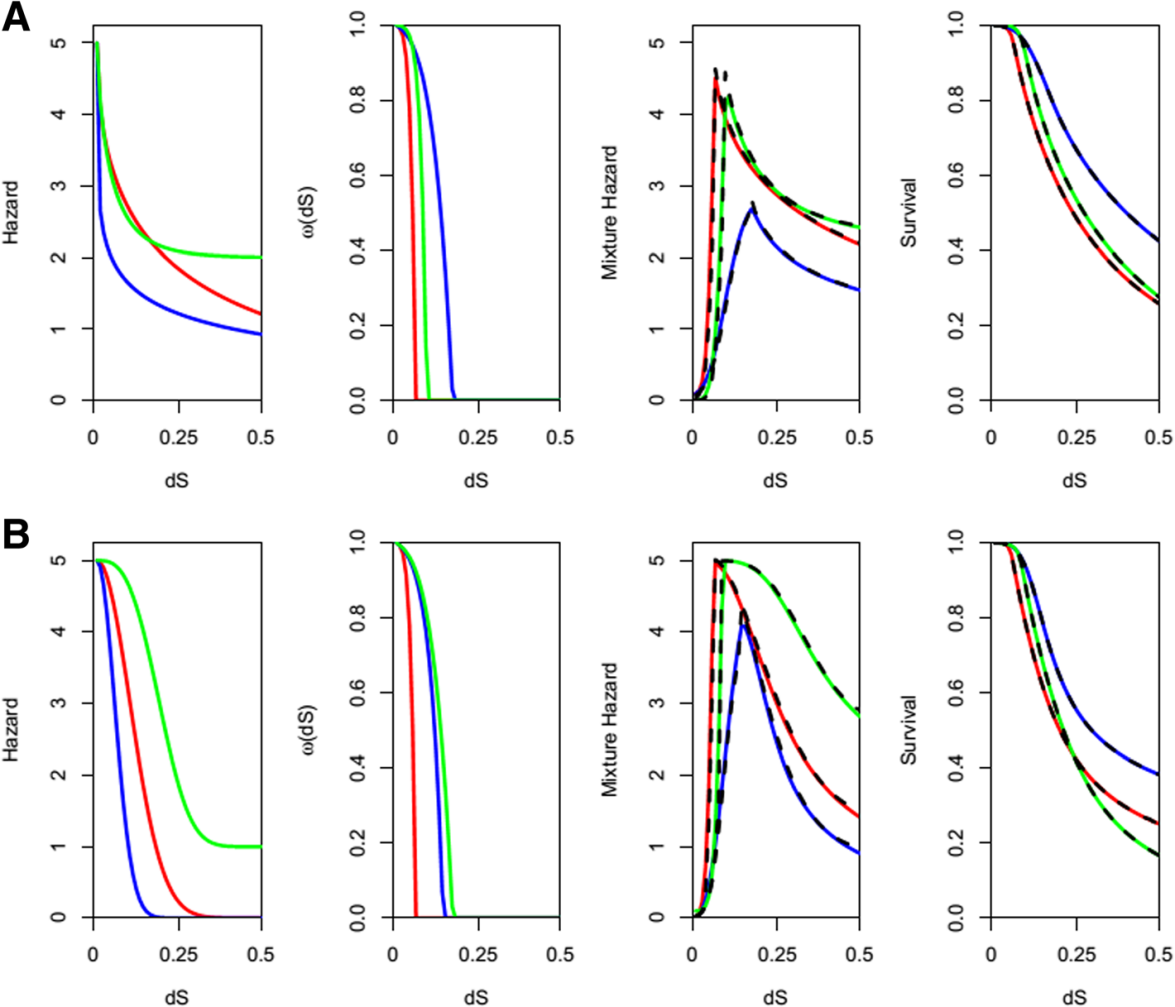
Comparison of integrative model and piece-wise model. Three different parameterizations of mixture processes of dosage balance and neofunctionalization (**a**) and subfunctionalization (**b**) produced using the weighted mixture model are shown. The survival data corresponding to these curves was fit using the piecewise model, and these fits are given in black dashed lines. From left to right, the plots show the neofunctionalization or subfunctionalization components of the hazard, the dosage balance component of the hazard, the mixed hazard function, and the corresponding survival function

The corresponding survival data was produced by evaluating the function

7 $$ S(t)={e}^{-{\displaystyle {\int}_0^t\delta (u)du}} $$

u, like x and y from eq. (3) is a variable of integration to track time and cumulatively integrates over mechanism transitions. Using this survival data, the survival function corresponding to the piece-wise model was then fit to this dataset. The parameters d, c, f, g, h, j, and k of the piece-wise model were simultaneously estimated by minimizing the sum of squares between the known survival data generated by the weighted mixing method and survival data proposed by the piece-wise model with the use of differential evolution [23]. Examples of the fit are given in Fig. 2 where the black dashed lines represent the best fitting piece-wise model and the corresponding parameter values are given in Table 1.

While the recovered parameters are not expected to match those under which the data was simulated because the generative model and the fitted model are different, the mechanisms under which the data was simulated are recoverable. Noticeably, the value of c estimated by the best fitting model is still consistent with the curve shape of the retention mechanism the data was simulated under. The recovered d + f values give the highest level of hazard experienced by duplicates undergoing retained by a mixture of processes. The g parameter is of interest because it gives an estimate of the x-intercept of *ω*(*t*), which gives the averaged length of evolutionary time duplicate genes were protected by dosage balance until they began to experience nonfunctionalization-like loss.

**Fig. 3:**
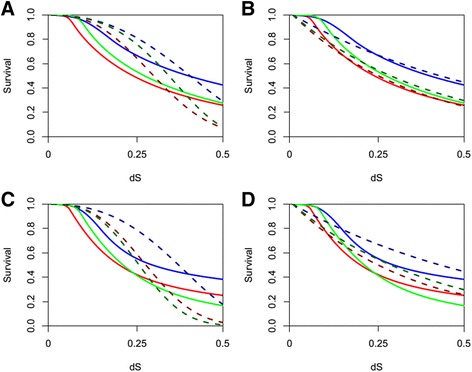
Comparison of model fit to Konrad et al. 2011. Using the models outlined in [13], which described the dynamics of duplicate gene retention due to a single mechanism, the dosage balance model (**a**) and neofunctionalization model (**b**) are fit to the survival data given in Fig. 2a. The dosage balance model (**c**) and subfunctionalization model (**d**) are fit to the survival data given in Fig. 2b. Fitted models are delineated by dashed lines and are given in darker hues of the corresponding survival data

To demonstrate the necessity of considering the hybrid retention processes when examining duplicate gene survival data, the dosage balance, neofunctionalization, and subfuctionalization models outlined in [13] (which each only account for a single retention mechanism) were fit to the datasets shown in Fig. 2. Figure 3 demonstrates the poor correspondence of data generated from models for multiple retention mechanisms fit to models which assume the dynamics of the loss process are due to only a single mechanism of retention. It is only with these new mixed (hybrid) models that the more complex processes that treat dosage balance as a transition state to eventual changes in function of the gene can be accurately captured statistically. Although model mis-specification was not evaluated here (through formal model selection with competing models), not only is the fit poor in Fig. 3b, d, but there remains the potential to interchange neofunctionalization and subfunctionalization as single model fits when they are processes acting together with dosage balance. When the dosage balance model is fit to the data generated under a mixture of processes (Fig. 3a, c), the resulting fit of this model is visually even worse, and while the mechanism is unlikely to be mis-specified if it is the supported model, the opportunity to identify cases of neofunctionalization is lost. It is therefore only with the new models presented in this work that the combination of neofunctionalization or subfunctionalization with dosage balance can be detected using the framework of Konrad et al. [13].

### Incorporation of the model in a phylogenetic birth-death model

The hazard function (3) can be incorporated into the age-dependent birth-death model [21] to find the likelihood function of duplication times. Specifically, the mean loss rate at time *t* is *ϕ*_*t*_ = *E*(*δ*(*t*_*i*_^'^)) = ∫_0_^*t*^*δ*(*t*_*i*_^'^)*f*(*t* ')*dt* ', in which *f*(*t* ') is the density function of gene age *t* ' (see Eq. 6 in [21]). In addition, the probability *P*(*τ*, *T*) that one lineage at time τ leaves multiple descendants at the present time *T* is given by *P*(*τ*, *T*) = [1 + ∫_*τ*_^*T*^*ϕ*_*t*_*e*^*β*(*τ*,*t*)^*dt*]^− 1^ and *β*(*τ*, *T*) = ∫_*τ*_^*T*^(*ϕ*_*s*_ − *γ*)*ds*, in which *γ* is the constant duplication rate. Let *u*_*ij*_ be the probability *P*(*n*_*j*_ > 1 | *n*_*i*_ = 1) that one lineage at time *t*_*i*_ leaves multiple descendant reconstructed lineages at a later time *t*_*j*_. This probability has been derived under the birth-death model, i.e., ${u}_{ij}=P\left({n}_j>1\Big|{n}_i=1\right)=1-P\left({t}_i,{t}_j\right){e}^{\beta \left({t}_i,{t}_j\right)}$ (see [24]). Given the number *n*_*T*_ of lineages at the present time *T* and the number *n*_0_ of lineages at time 0, the joint density function of the duplication times *t* = {*t*_*i*_ | *i* = *n*_*0*_ + 1, …, *n*_*T*_} is given by [21].

$$ f\left(t\Big|{n}_T,{n}_0,T\right)=\frac{{\displaystyle {\prod}_{i={n}_0+1}^{n_T}}\left(i-1\right)\gamma P\left({t}_i,T\right){\left(1-{\eta}_{t_{i-1},{t}_i}\right)}^{i-1}}{\left(\begin{array}{c}\hfill {n}_T-1\hfill \\ {}\hfill {n}_0-1\hfill \end{array}\right){\left(1-{\eta}_{0,T}\right)}^{n_0}{\eta}_{0,T}^{n_T-{n}_0}}, $$

In the joint density function, ${\eta}_{ij}=1-\frac{1-{u}_{iT}}{1-{u}_{jT}}$. This joint density function can be used as the probability distribution of branch lengths of the gene family tree, when analyzing the sequences of gene family data.

## Conclusions

Duplicated genes serve as an important contribution to drive functional and structural divergence in genomes. The model introduced here builds on a framework initially given in [13], and represents another step in building more complex, more realistic mechanistic models to characterize the retention patters of duplicated genes. While further work is needed to fully characterize the process of duplicate gene retention and loss, such as a realistic model of gene birth, the work presented here can ideally be extended as part of a birth-death model for use in a gene/tree species tree reconciliation context [21]. Including further levels of biological reality in methods for gene/tree species tree reconciliation should not only increase the accuracy of estimates for the timing and evolutionary history of genes but can also offer insight into how genes and genomes evolve. One such extension includes the characterization of the underlying population genetics of fixation as a loss/retention process, given that genes are typically sampled from a single individual from a species, and the associated probabilities can now be incorporated into this model [25]. As these models and modeling frameworks converge, comparative genomics will have a powerful toolbox with which to make probabilistic characterizations of mechanisms of duplicate gene retention, where for example indications of neofunctionalization may be useful in predicting lineage-specific functional change between closely related species.

### Availability of supporting data

This work is of a theoretical nature and no new data was generated for this manuscript.
